# Trainee’s Perceptions and Practices on Social Media at One Internal Medicine Program and Its Potential Uses in Graduate Medical Education

**DOI:** 10.15694/mep.2019.000116.1

**Published:** 2019-05-30

**Authors:** May Lee, Cha-Chi Fung

**Affiliations:** 1 Keck School of Medicine at the University of Southern California

**Keywords:** social media, medical education, physicians, education technology

## Abstract

This article was migrated. The article was marked as recommended.

Introduction:

Social media is a potential tool in Graduate Medical Education (GME). However, the perceptions that trainees themselves hold about the usefulness of social media in education has not yet been examined.

**Methods**:

A 34-item questionnaire was conducted at LAC+USC Medical Center in Los Angeles, CA and distributed to all Internal Medicine (IM) and Medicine-Pediatric residents and IM subspecialty fellows September through December 2017 with a 31% response rate. Most questions utilized 5-point or 6-point Likert-type scales.

**Results**:

We found that IM GME trainees were familiar with, and used social media regularly, with Messaging apps and social networks used most frequently. Video content/sharing and podcasts were identified as having the most potential for education. Trainees were in strong agreement about openness to social media use in GME. However, the majority of respondents answered “maybe” when asked whether it would be a positive addition. The majority of trainees did not see social media as unprofessional, and were mixed about whether or not it would be an invasion of privacy.

**Conclusions**:

Our analysis of the data reveals that social media may be a potential tool, but ambiguity remains as to whether or not social media tools would be fully embraced by trainees.

## Introduction

Social media has become a ubiquitous part of daily life. It has changed the way we communicate, obtain and disperse information, and interact. It has been shown in the education literature that social media has the ability to increase engagement and interest in the subject matter being studied. ( Cunha, Van Kruistum, and Van Oers, 2016) By its nature, social media is collaborative. This collaborative nature lends itself to the ability to facilitate ongoing conversations where learners can continue to learn and engage outside the classroom. In addition, because of the adaptable, customizable, and immediate nature, social media can address several tenets of adult learning theory (1) relevancy-oriented and practical information (2) active participation in the learning process (3) timely feedback and reinforcement of learning (4) informal and personal learning environment ( Collins, 2004). For this generation of learners, technology is integrated into daily life. To date, there have been multiple published examples of undergraduate, graduate and even some Graduate Medical Education (GME) programs that have used social media tools for teaching purposes. ( Bogoch *et al*., 2012) ( Granitz and Koernig, 2011) ( Pearson *et al*., 2016) ( Shah and Kotsenas, 2017) ( Sterling *et al*., 2017) ( Saeed, Yang, and Sinnappan,S., 2009) A systematic review of social media use in medical education published in 2013 showed that “the opportunities related to incorporating social media tools were promoting learner engagement (71% of studies), feedback (57%), and collaboration and professional development (both 36%).” ( Cheston, Flickinger, and Chisolm MS, 2013)

While we may presume that social media is universally accepted in this current generation, there is some research to show that the so-called “digital natives” may not be uniform in their acceptance and use of technological tools in education, and that their use of social media is for primarily interactive and informational purposes that are not education driven. ( Neier and Zayer, 2015) Additionally, a study by Killan *et al.* showed that the millennial generational is not monolithic in the ways in which they use social media. They were able to categorize them into “the restrained millennials,” the entertainment seeking millennials,” and the “highly connected millennials” and identified that these groups used the same social media in different ways (i.e. passively or actively). ( Kilian, Hennings, and Langner, 2012)

Given that there are potential educational opportunities for social media in medical education and that there is variability in use of this media in this generation of trainees, it is important to examine the perceptions the trainees themselves hold about its usefulness in enriching their learning environment. By understanding the useage, the motivations and the acceptability of social media we sought to answer the question as to whether or not social media could be adopted as a learning tool in GME, and if so, which platforms would be best accepted.

## Methods

We developed a 34-item questionnaire that was based on a validated 47-item survey instrument from work published by and with permission from Neier and Zayer, 2015. Our 34-item questionnaire also included 4 demographic variables (gender, age, level of training, and self-described ethnic group). Most of the questions utilized 5-point or 6-point Likert-type scales. The surveys were pilot tested with Pulmonary and Critical Care Medicine fellows prior to the final distribution to the rest of the Internal Medicine (IM) trainees.

This survey study was conducted at LAC+USC Medical Center in Los Angeles, CA from September through December 2017 and distributed to all the Internal Medicine (IM) and Medicine-Pediatric residents as well as IM subspecialty fellows. The questionnaire was distributed anonymously through Qualtrics.

The Institutional Review Board at the University of Southern California approved the study as an exempt study in September 2017.

Statistical analysis was performed using SPSS and Excel to tabulate frequencies and descriptive statistics. Chi-Square testing was used to cross tabulate demographic information and responses to specific questions.

## Results/Analysis

Of the 312 surveys distributed, there were 96 responses (31%). There was, however, significant dropout towards the end of the survey, resulting in missing demographic data in about 33% (64 of 96 completed, 66%). Table 1 describes the characteristics of survey participants.

**Table 1.** Participant Characteristics

Parameter	n	percent
Level of Training		
Fellow Year 1	8	12.5
Fellow Year 2	13	20.3
Fellow Year 3	6	9.4
PGY1	10	15.6
PGY2	8	12.5
PGY3	15	23.4
PGY4	4	6.3
Age		
25-29	26	40.3
30-34	37	58.1
35-39	1	1.6
Gender		
male	37	57.8
female	27	42.2

Familiarity: ( Table 2) When asked which platforms trainees were most familiar with (1= not at all familiar; 2=slightly familiar; 3=moderately familiar; very familiar; 5 = extremely familiar), they were most familiar with messaging apps (mean [x] = 4.47, standard deviation [SD] = 0.67), social networks (x = 4.38, SD = 0.80), and video content/sharing (x = 4.21, SD = 0.91). Trainees were least familiar with pinning sites (x=2.47, SD=1.22) and microblogging (x=2.48, SD=1.03)

**Table 2.** Familiarity with Platforms (n=92)

Platform	*x̄*	SD
Social network (Facebook)	4.38	0.80
Professional focused social networks (Linkedin)	2.84	1.14
Microblogging (Twitter)	2.48	1.03
Messaging app (What’s App, Viber)	4.47	0.67
Multimedia (Snapchat)	2.97	1.44
Pinning sites (Pinterest)	2.47	1.22
Video content and sharing (Youtube)	4.21	0.91
Photo sharing sites (Instagram)	3.80	1.21
Podcasts	3.07	1.13

1= not at all familiar; 2=slightly familiar; 3=moderately familiar; very familiar; 5 = extremely familiar

Usage ( Table 3): Given the Likert scale 1-6; 1=never; 2= at least once in my life; 3= at least once a month; 4= at least once a week; 5= at least once daily; 6= multiple times a day, messaging apps were used most often (x=5.76, SD=0.85). Social networks were used at least once daily (x=5.05, SD= 1.31), and video content/sharing sites were used at least once weekly (x= 4.24, SD=1.60). Microblogging was used least often, averaging about once in a lifetime (x=1.85, SD=1.28)

**Table 3.** Usage (n=86)

Platform	x̄	SD
Social network (Facebook)	5.05	1.31
Professional focused social networks (Linkedin)	2.52	1.27
Microblogging (Twitter)	1.85	1.28
Messaging app (What’s App, Viber)	5.76	0.85
Multimedia (Snapchat)	3.87	1.88
Pinning sites (Pinterest)	2.18	1.40
Video content and sharing (Youtube)	4.24	1.60
Photo sharing sites (Instagram)	3.73	1.69
Podcasts	2.82	1.48

1=never; 2= at least once in my life; 3= at least once a month; 4= at least once a week; 5= at least once daily; 6= multiple times a day

Potential tools ( Table 4): When asked about their opinion on the platform with the most potential for education (1= no potential; 2=weak potential; 3=neutral; 4=moderate potential;5= strong potential), the top two were video content/sharing (x=4.43, SD=0.74) and podcasts (x=4.38, SD=0.80). Those with the least potential were identified as pinning sites (x=2.89, SD=1.07) and microblogging (x=3.15, SD=1.07)

**Table 4.** Potential for learning (n=72)

Platform	x̄	SD
Social network (Facebook)	3.52	1.09
Professional focused social networks (Linkedin)	3.49	1.14
Microblogging (Twitter)	3.15	1.07
Messaging app (What’s app, Viber)	3.60	1.10
Multimedia (Snapchat)	3.57	1.13
Pinning sites (Pinterest)	2.89	1.07
Video content and sharing (Youtube)	4.43	0.74
Photo sharing sites (Instagram)	3.71	1.14
Podcasts	4.38	0.80

1= no potential; 2=weak potential; 3=neutral; 4=moderate potential; 5= strong potential

How social media might enhance learning: The highest motivational drivers seemed to be attributed to interaction (x = 3.67, SD = 1.06), being informed (x = 3.59, SD = 1.00), and entertainment value (x = 3.48, SD = 1.10).

Agreement about openness to social media: Given the question “I am open to using social media tools in the classroom to enhance my learning experience?” when given the scale of 1=strongly disagree to 5= strongly agree, the mean was 3.96 (SD = 1.0). To the question, “I would like my faculty instructors to use social media tools in the classroom for teaching purposes,” the mean was 3.5 (SD = 1.01) and to the question “I would perceive my training program as innovative if my classes incorporated the use of social media tools for teaching purposes”, the mean was 3.71 (SD=1.09). In an open-ended question, those who expressed positivity towards using social medial as an educational tool cited “multimedia learning,” “interactive,” “faster communication” “innovative and engaging ways of learning” and “less formal setting” as positives. For those reluctant to use social media, they cited “separation of work and personal life,” “invasion of privacy,” “not meant to be used for education,” and “cannot replace hands on, person to person interactions” as reasons.

When asked if they felt “social media be a positive addition to the training program?” 42% responded “yes,” 53.2% responded “maybe” and 4.8% responded “no.”

Reluctance to use social media: The majority of trainees did not see social media as unprofessional (x=2.74, SD 1.10) using the scale 1=strongly disagree to 5= strongly agree. They were somewhat more mixed when asked whether or not they saw the use of social media in the learning environment as an invasion of privacy (x=3.13, SD=1.07).

**Figure 1.**
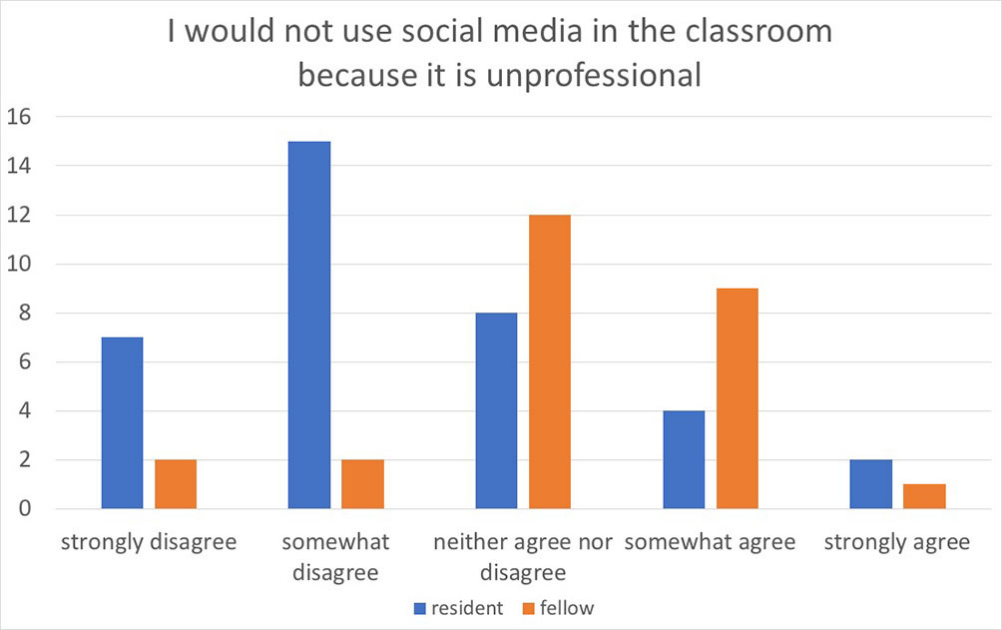
Resident versus Fellow Perception of Social Media Unprofessionalism

**Figure 2.**
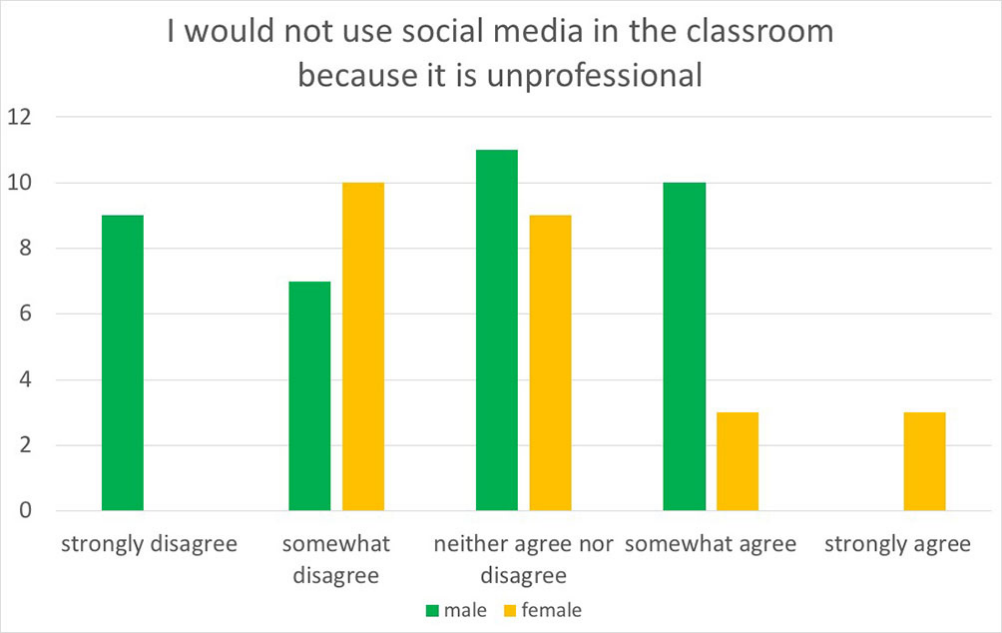
Male versus Female Perception of Social Media Unprofessionalism

We also cross-tabulated demographic data with the three questions: “social media would be a positive addition to the training program,” “I would not use social media in the classroom because it would invade my privacy,” and “I would not use social media in the classroom because it is unprofessional.” There was no difference in comparing the demographic groups when asked about agreement with the first two statements. However, when comparing the agreement with the statement “I would not use social media in the classroom because it is unprofessional”, we found a statistically significant association when comparing two groups. Fellows were more in agreement than residents (X ^2^=14.541, p=0.006) [ figure 1] and females were more in agreement than males (X ^2^=14.728, p=0.005) [ figure 2].

## Discussion

While social media is used almost ubiquitously in society,the perceptions that Internal Medicine GME trainees themselves hold about the usefulness of social media in education has not yet been examined. We set out to ascertain whether these tools are appropriate for use in GME, and whether these tools will be accepted in this population, and evaluate which platforms might be best suited for GME use. In this study of social media practice and perceptions in IM trainees at the LAC+USC training program, we found that although social media is used frequently, trainees are somewhat cautious about its use in medical education. We found that most trainees actively use social medial in their daily lives, with many interfacing with some form of social media several times a day. This usage and familiarity, however, may not translate into direct use for educational purposes. In addition, the highest usage platforms (social networks and messaging apps) were not found to be the same as those seen as most useful for education (video content/sharing and podcasts). The least used platform correlated with the platform seen as having the least potential for education (microblogging).

Our analysis of the data also reveals that not all trainees may uniformly accept social media tools in the classroom. If social media is used, careful consideration of acceptable platforms, as well as the motivations for use should align.

In addition, although trainees indicated that they are open to using social media tools in the classroom to enhance learning and see its use as innovative, they are still somewhat mixed as to whether or not the use of social media in education would be a positive addition. This reluctance may be due to concerns of privacy. While trainees as a whole did not see social media as unprofessional, we found that fellows and females were more likely than residents and males to see social media as being unprofessional, perhaps indicating that social media as an educational tool may be more acceptable to male trainees and those who are earlier in training.

A major limitation of this study was the response rate (31%) as well as the eventual completion rate of the survey (64 of 96 completed, 66%). Many of the comments in the pilot survey related to the survey noted the length and redundancy of the instrument. As a result, 10 questions were omitted in the final questionnaire; however, we likely could have shortened it more to increase the completion rate. Another potential limitation is that this study was distributed to trainees in Internal Medicine at one training program. Students’ perceptions of social media in education could be affected by the type of training program, as well as geographic location or institution.

## Conclusion

We find that while IM GME trainees are open to use of social media in medical education, they are unclear whether it would be positive addition to their training, and there was some reluctance due to privacy concerns. The platforms that seem most acceptable to this population are video content/sharing and podcasts. While there is still some ambiguity as to whether or not social media tools would be fully embraced, there is room for further study. Trials using specific platforms should be undertaken with analysis of its effectiveness and acceptability amongst trainees.

## Take Home Messages

• While there is much interest in using social media in graduate medical education, the perceptions the trainees themselves hold about its usefulness has not been previously studied.
• While IM GME trainees are open to using social media in their education, they are uncertain whether it would be positive addition to their training.
• Video content/sharing and podcasts seem to be the platforms most acceptable to this population.
• There is room for further study whether or not social media tools would be fully embraced.
• Trials using specific platforms should be undertaken with analysis of its effectiveness and acceptability amongst trainees.

## Notes On Contributors

Cha-Chi Fung, Ph.D. is the Vice Chair of the Department of Medical Education, Assistant Dean of Educational Affairs and Associate Professor in the Department of Medical Education at the Keck School of Medicine of the University of Southern California.May Lee, MD is the Program Director for the Pulmonary and Critical Care fellowship program and Assistant Professor of Medicine in the Department of Medicine at the Keck School of Medicine of the University of Southern California.
