# Supplementary material for: Trainee’s Perceptions and Practices on Social Media at One Internal Medicine Program and Its Potential Uses in Graduate Medical Education
Source: MedEdPublish (2016). 2019 May 30;8:116. [Version 1] doi: 10.15694/mep.2019.000116.1 (PMC10712522; doi:10.15694/mep.2019.000116.1)
Supplement: Peer review history

# Reviewer report: Reviewer response for version 1

**DOI:** 10.21956/mep.19847.r29939

**Published:** 2019-08-20

**Authors:** Poh-Sun Goh

**Affiliations:** 1 National University of Singapore

This review has been migrated. The reviewer awarded 4 stars out of 5 This paper is clearly written, and adds to the literature on this topical subject.The introduction section, with references cited is recommended to readers who would like to get an overview of, and read further on this subject. For example, one of the papers cited, and referenced by the authors (see below also) is a useful place to start.Pearson, D., Cooney, R. and Bond, M. (2015) ‘Recommendations from the Council of Residency Directors (CORD) Social Media Committee on the Role of Social Media in Residency Education and Strategies on Implementation,’ Western Journal of Emergency Medicine, 16(4), pp. 510–515. https://doi.org/10.5811/westjem.2015.5.25478It would have been useful for the authors to have provided further information about how social media has been used in professional practice and training in their study cohort, and specifically how social media has been integrated into the training programs of the survey cohort. Incorporating the survey instrument as part of the program evaluation process would be anticipated to have significantly increased the participation rate for this research study.It would also be useful to read about the authors planned next steps following this study, and also to hear about how the authors might address the following training challenges - To use selected social media, and how to integrate this into their professional training programs. Should this be instructor led, resident and fellow led, or part of a community of practice?How important is content quality, relevance, and usefulness to the magnitude of participation rates (in use of social media in professional training)?

Reviewer Expertise:

NA

No decision status is available

# Reviewer report: Reviewer response for version 1

**DOI:** 10.21956/mep.19847.r29941

**Published:** 2019-06-13

**Authors:** Anna Greer

**Affiliations:** 1 University of Sheffield

This review has been migrated. The reviewer awarded 4 stars out of 5 This is a topical article that asks some important questions about the use of social media in graduate medical education. Whilst the questions asked in the survey were all relevant, the addition of a qualitative component could have created opportunities to better understand the survey answers. The original use of the survey (by Neier and Zayer) was supplemented by in-depth interviews. As acknowledged by the authors, the main limitation was the response rate. This is often a frustrating part of using a survey to collect data, but no information was provided on how often the survey was distributed during the study period. This is an interesting article that opens many more questions about the use of social media in graduate medical education as well as highlighting some of the issues faced by educators.

Reviewer Expertise:

NA

No decision status is available

# Reviewer report: Reviewer response for version 1

**DOI:** 10.21956/mep.19847.r29940

**Published:** 2019-05-30

**Authors:** Sateesh Babu Arja

**Affiliations:** 1 Avalon University School of Medicine

This review has been migrated. The reviewer awarded 4 stars out of 5 It is an interesting paper in the current situation of medical education. The current generation of medical students and trainees are very much interested in technology-enhanced earning. Any strategy or method of learning will not be fully successful or achieve desired outcomes until unless it is fully embraced by students or trainees. It is very interesting to see podcasts scored higher than LinkedIn or Twitter in terms of familiarity, usage, and potential for learning. Podcasts can be a great platform for learning as they are of optimal length (10-25 min), delivered effectively and engaging the learners. As authors quoted one of the limitations for this paper is low response rate. This manuscript could be useful for medical educators and medical education units.

Reviewer Expertise:

NA

No decision status is available
